# Antifungal Potential and Antioxidant Efficacy in the Shell Extract of *Cocos nucifera* (L.) (Arecaceae) against Pathogenic Dermal Mycosis

**DOI:** 10.3390/medicines3020012

**Published:** 2016-05-25

**Authors:** Nasreen Khalid Thebo, Altaf Ahmed Simair, Ghulam Sughra Mangrio, Khalil Ahmed Ansari, Aijaz Ali Bhutto, Changrui Lu, Wazir Ali Sheikh

**Affiliations:** 1 College of Chemistry, Chemical Engineering and Biotechnology, Donghua University, Shanghai 20160, China; crlu@dhu.edu.cn; 2 Mycology Research Laboratory Institute of Plant Sciences, University of Sindh, Jamshoro 76080, Sindh, Pakistan; nasreenthebo@gmail.com (N.K.T.); wazirshaik16@hotmail.com (W.A.S.); 3 Department of Biotechnology, Sindh Agricultural University, Tandojam 70060, Sindh, Pakistan; sughra_m@hotmail.com; 4 Department of Botany, Shah Abdul Latif University, Khairpur Mir’s 66111, Sindh, Pakistan; khalilahmedansari@hotmail.com; 5 National centre of Excellence in Analytical chemistry, University of Sindh, Jamshoro 76080, Sindh, Pakistan; bhuttoaijaz2008@gmail.com

**Keywords:** coconut shell, Soxhlet extraction, phenolic constituents, DPPH antioxidant process, antifungal activity

## Abstract

**Background:** Coconut is a tropical fruit well known for its essential oils that have been recognized for their biological activities since ancient times. There have been no previous investigations on the essential oils from coconut shells. **Method:** The shell extract of *Cocos nucifera* (L.) was prepared by the Soxhlet method and total phenolic content (TPC) in the extract was determined by Folin-Ciocalteu (FC) assay. The antioxidant potential of the coconut shell extract was evaluated by using the 2,2-diphenyl-1-picrylhydrazyl (DPPH) radical scavenging assay. Minimum inhibitory concentration (MIC) of the extract was determined by the strip method against clinically isolated dermal mycosis of 20 infected patients. **Result:** Total antioxidant activity varied from 92.32% to 94.20% and total phenolic content was found at 5.33 ± 0.02 mg/g in the coconut shell extract. The extract was found to be most effective as an antifungal against human pathogenic fungi, including *A. niger*, *A. flavus*, *T. rubrum*, *M. canis*, *M. gypseum*, *A. fumigates*, *T. mentagrophyte* and *T. vercossum*. The crude shell extract was highly effective against all dermal mycosis tested with the MIC ranging from 62 mm to 90 mm, whereas all fungal samples showed good inhibitory effect. **Conclusion:** The results of the present study provide a potential cure for microbial infections.

## 1. Introduction

Coconut is a tropical fruit largely consumed in many countries for its nutritional and medicinal properties. Coconut has also been recognized since antiquity as a source of essential oils displaying biological activities [1]. Many coconut products are used in various parts of the world; especially in coastal areas, 60% of the domestic waste corresponds to coconut shell [2]. Studies have been reported that different parts of the coconut are used as traditional medicines in different regions of the world for different diseases and as preventative alleviation of symptoms related to menopause [3,4,5]. The demand for phenolic extracts has increased due to their potential applications in food and pharmaceutical industries. Plants possess antimicrobial and antioxidant activities responsible for the prevalence of dermatophytosis commonly known as *Tinea* infection. In 1977, when Emmons *et al.* [6] reported that fungal species *Microsporum*, *Epidermatophyton* and *Trichophyton* are causative agents for the mycotic infection, a great deal of attention was drawn to the use of industrial plant-derived fungicides based on the flora. It is an old practice to use fungicides as defense against skin diseases, including mycotic transmission, in many parts of the world [7]. Coconut water and milk are also used as an antiulcer treatment and food supplement due to its high nutritional value [8]. At present, the use of agricultural waste is receiving more attention than in the past for the production of natural products because these are cheaper, highly effective and the best solution for the disposal of waste. Phenolic compounds have high antioxidant potential and antimicrobial properties and are good food stabilizers [9]. The antioxidants in foods of plant origin inhibit scavenging of free radicals, chelate compounds, metallic elements and lipoxygenase [10]. Several fruits have been characterized for their phenolic profile and antioxidant activity [11,12]. The medicinal properties of the coconut plant have been attributed to the presence of these biologically and chemically active features. The pharmacologically active molecules, propolis, flavonoids and phenolic acid components are present in almonds and other nuts, giving them a beneficial dietary character through the phenolic components [13,14]. Although many studies have been undertaken on different parts of coconut, the shell has not yet been the focus of research. In this study, we have used for the first time coconut shell extract for the evaluation of antioxidant and antifungal potential and therapeutic efficacy against clinically isolated dermatomycosis.

## 2. Material and Methods

### 2.1. Shell Sample and Reagents

**Figure 1:**
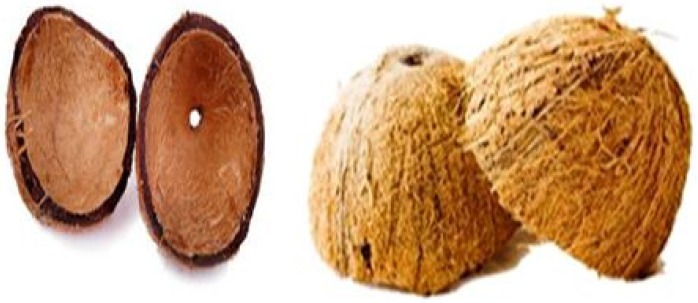
*Cocos nucifera* (Coconut) sample.

Coconut (*Cocos nucifera*) sample was collected from the local market of Hyderabad (Figure 1). The 2,2-diphenyl-1-picrylhydrazyl (DPPH), Folin-Ciocalteu phenol reagent, sodium carbonate and gallic acid were purchased from Sigma-Aldrich (St. Louis, MS, USA). Dextrose agar and all other chemicals and reagents were used of analytical grade. The solution preparations were made using double distilled water. The absorbance was evaluated using the lambda 35 UV-Vis spectrophotometer (Perkin Elmer, Richmond, VA, USA).

### 2.2. Soxhlet Extraction of Coconut Shell

Coconut shell fibers were separated and powdered using Waring blender (Cixi Lotek Electrical Appliance Co., Ltd., Zhejiang, China). A total of 5 gm of shade-dried powder was filled in a thimble and subsequently extracted with methanol using the Soxhlet extractor for 44 h. The solvent extract was filtered through Whatman No. 1 single filter paper to remove waste and rough particles. The crude extract was stored at 18 °C until analyzed.

### 2.3. Solvents Extraction for Antifungal Assays

The collected plant material was washed with distilled water and placed in the shade at room temperature for two weeks. Approximately one kilogram of dried plant material was soaked in the bottle containing 2 L of ethanol for 20 days. The extract was then filtered using a rotary evaporator under reduced pressure below 40 °C. The concentrated residue was completely dry and converted into powder. The residue was extracted in a separating funnel using four different solvents including ethyl acetate, chloroform, methanol and distilled water. The extracts were left at room temperature to remove solvent from the samples. Antifungal activity was observed for each extract reported by Harborne, *et al.* [15].

### 2.4. Saponification

An amount of 150 mL ethanol (EtOH) and water (H_2_O) (1:1, *v*/*v*) containing 10% KOH was added to the residue, and the reaction mixture was refluxed at 100 °C for 6 h. The mixture was concentrated under reduced pressure using a rotary evaporator before water (H_2_O) and diethyl ether (Et_2_O) were added. The procedure was repeated three times. The un-saponifiable matter was partitioned and separated. The aqueous alkaline fraction was acidified with 6N HCl (pH 5–6) and extracted several times with (Et_2_O). The total Et_2_O fraction was dried over anhydrous Na_2_SO_4_ and, upon evaporation of Et_2_O, residue was obtained.

### 2.5. Collection of Dermatophytes

**Figure 2:**
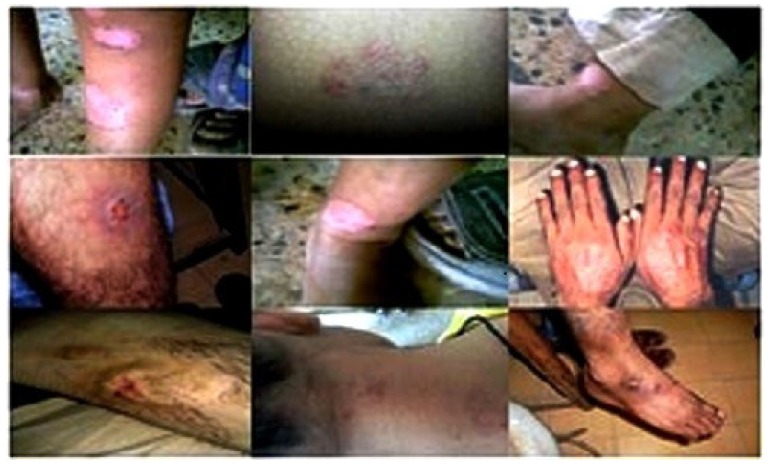
Clinical presentation of host reaction to the more common mycoses *Tinea corporis, Tinea manum, Tinea cruris* and *Tinea pedis.*

**Figure 3:**
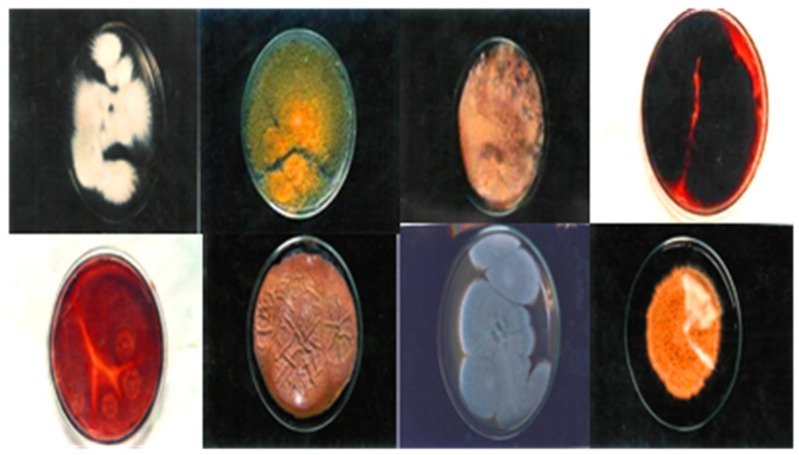
Clinically isolated dermal mycosis (positive control), 138" × 2.71".

**Figure 4:**
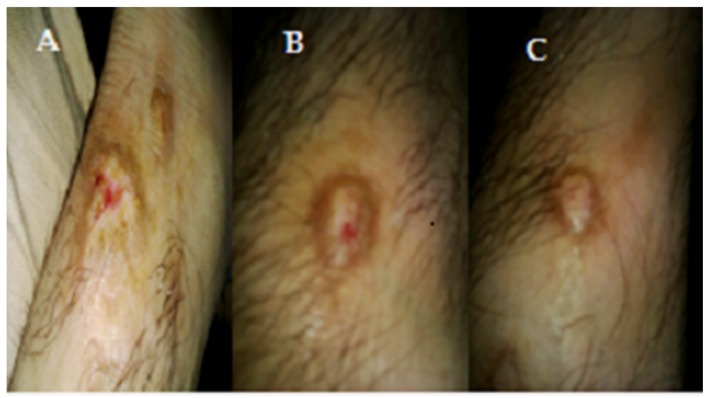
(**A**) Before treatment, *Tinea corporis* infection of patient; (**B**) After 1 week of treatment of shell extract on *Tinea corporis* infection of patient; (**C**) After 2 weeks of treatment of shell extract on *Tinea corporis* infection of patient, 1.73" × 3.11.

The antidermal activities were evaluated against *Aspergillus niger, Microsporum canis, Microsporum gypseum, Aspergillus flavus, Trichophyton rubrum, Aspergillus fumigatus* and *T. vercossum*. Mycosis clinically isolated from *Tinea corporis* (ringworm)-infected patients as shown in Figure 2, Figure 3 and Figure 4.

The crude extract was individually tested against dermal mycosis. All fungal cultures were observed for the minimum inhibitory concentration (MIC) using the strip method. The patient was observed clinically at the skin department of Liaquat University of Medical and Health Sciences, Hyderabad, Sindh, Pakistan. The samples were collected by scraping the superficial infected skin from the *Tinea corporis* infection.

### 2.6. Preparation of Fungal Culture for Antifungal Screening

Different solvent fractions of the coconut shell extract were individually tested against eight dermal pathogenic fungi. All fungal cultures were tested for minimum inhibitory concentration (MIC). Sabouraud dextrose agar (SDA) was used to grow the fungi and all solutions were autoclaved at 121 °C, 15 lb/inch² pressure for 20 min.

### 2.7. Antifungal Screening

The minimum inhibitory concentration (MIC) values were confirmed for pathogenic fungi by the strip method and found to be sensitive to the shell extract of *Cocos nucifera*. The SDA medium was placed on 96-well plates for healthy growth of culture, and zones of inhibition MIC were carried out using different stock solution concentrations and serial dilutions. MIC was determined by the strip dilution method. A dilution series was set up by using graded concentrations for four replicates for each experiment for each fungal sample [16].

### 2.8. Antioxidant Activity

The shell extract of *Cocos nucifera* was tested for antioxidant potential using the 2,2-diphenyl-1-picrylhydrazyl radical (DPPH) assay method reported by [17]. Freshly prepared solution of DPPH (0.5 mL) was added to 1.0 mL of shell extracts. The decrease in absorbance was measured at different time intervals, *i.e.*, 0.05, 1, 2, 5 and 10 min (up to 40%). The decrease in the absorbance was determined at 515 nm, when the reaction reached a plateau. Gallic dose was taken as a standard antioxidant and observance of the DPPH radical without any antioxidant was measured as control [18,19].

### 2.9. Determination of Total Phenolic Content (TPC)

The total phenolic content was determined by using the Folin-Ciocalteu assay [9]. An aliquot (200 μL) of the diluted shell extract was added to 800 μL freshly prepared diluted Folin-Ciocalteu reagent and 2 mL of 7.5% Na_2_CO_3_. The final mixture was diluted to 7 mL deionized water and mixtures were kept in the dark at ambient conditions for 2 h to complete the reaction. After incubation for 2 h, absorbance against the reagent blank was determined at 765 nm with PerkinElmer LAMBDA-2 spectrophotometer. Gallic acid was used as standard and results were calculated as gallic acid equivalents (100 g of the seed coat). The reaction was conducted in triplicate and the results were averaged [20,21].

### 2.10. Statistical Analysis

Three replicates of each sample were used for statistical analysis. Data were reported as means ± S.D. Analysis of variance and least significant difference tests were conducted to identify differences among means. *t*-test was performed to determine significant differences at *p* < 0.05.

## 3. Results and Discussion

### 3.1. Extract Yield Estimation

Soxhlet extraction of coconut (*Cocos nucifera*) was carried out with methanol that exhibited the highest yield of extract. The highest yield in methanol may be hypothesized due to matching different origins of the two varieties, the environmental factor and the polarity of antioxidative compounds in coconut (*Cocos nucifera*) extracts and methanol.

### 3.2. Total Phenolic Content and Antioxidant Activity

**Figure 5:**
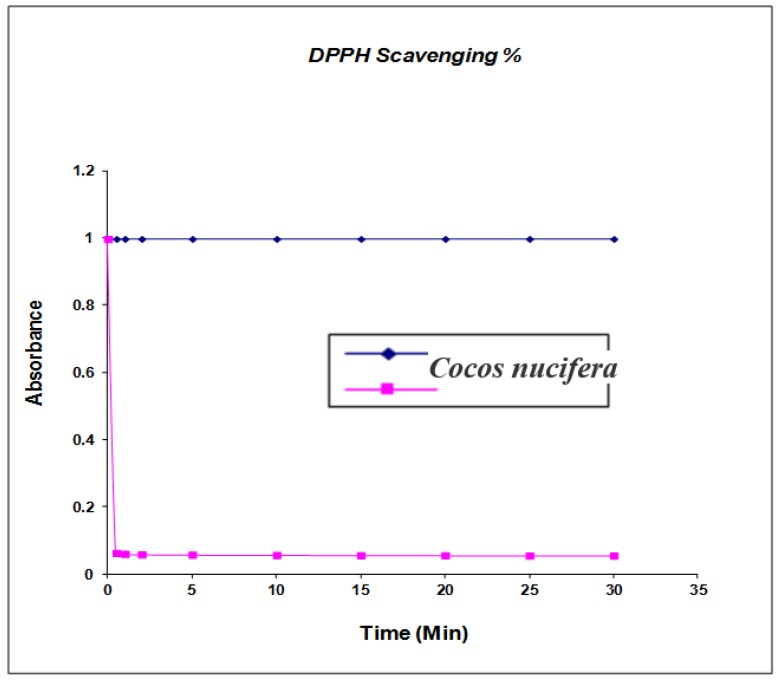
Antioxidant Activity and kinetic behavior or radical scavenging activity of *Cocos nucifera* extracts assayed by the DPPH method. The final DPPH concentration was kept at 100 μM in all reaction mixtures. Values are mean (*n* = 3), (*p* < 0.05).

The phenolic compounds may contribute directly to antioxidant activity [22] (shown in Figure 5); therefore, it is necessary to investigate the TPC in the extract from different solvents. Total phenolic contents were determined by Folin-Ciocalteu reagent and the results were expressed as gallic equivalents. Gallic calibration ranging from 0.002 to 0.008 mg/g was prepared and the results were determined from the regression equation of the calibration curve (*y* = 1786 *x* − 0.0047, *R* = 0.008) and were expressed as mg gallic acid equivalents (GAE). Total phenol contents were in the range of 3.11 ± 0.03 mg/g of coconut (*Cocos nucifera*) extract, whereas Fukuda *et al.* identified eight phenolic compounds in the minor shell with *p*-coumaric acid as the major chemical compound at 11.56% and five phenolic compounds in coconut seed with rutin trihydrate as the major compound at 4.32% [23]. The antioxidant efficacy of nut shell extracts was investigated by using 2-diphenyl-1-picrylhydrazyl. The DPPH assay is an established method for determining antioxidant potential and is based on measuring the scavenging capacity of antioxidants towards the stable free radical, 2,2-diphenyl-1-picrylhydrazyl, such as through the hydrogen-donating ability of phenolic compounds [24]. Kinetic studies of DPPH extracting the reaction were carried out by estimating binding activity as a function of time. The maximum difference between the extract was determined from calculating the difference between the beginning amount and the remaining amount (%) of DPPH after 5 min. Solar *et al.* confirmed that the ethanol extract with the highest percentage of DPPH binding activity had the lowest rates in dichloromethane [25]. In further studies, caffeic compound dihydroxyphenylacetic acid, syringic acid, *p*-coumaric acid, rutin trihydrate, nephtoresorinol, trans-2-dihydroxycinamic acid and dihydrate quercetin were also confirmed by Tejano *et al*. isolating these contents from nut tree green husks, green nut fruit leaves of inner sapwood, and waste tissues [26]. The average of superoxide radical scavenging percentage in four varieties of coconut hull phenolic extract (34.55% ± 1.14%) was obtained and maximum percentage (43.31% ± 1.48%) was recorded. The reducing power values in three coconut hull phenolic extracts was 0.212 AU at 700 nm [27,28]. Due to the above promising results it is necessary to confirm more phenolic compounds from coconut shell. The average reducing power value (0.148) in three wild coconut shell phenolic extracts was achieved by Cosmulescus *et al.* [29]. The previous studies reported that the average values in coconut hull extract (0.111) were higher than in their shell extract (0.134). Fruits are potential sources of natural phenolic antioxidant that can be used as a nutrient for health-promoting purposes The phenolic compound may contribute directly to antioxidative action [20]. Significant difference has been presented in TPC content among the samples. Kahkonen *et al.* [30] found TPC in the range of 2.51–3.5 mg/g for antioxidant activeness for the *n*-butanol extract of coconut. In the study of total phenolic contents of the extract of pine sawdust and coconut pulp, phenolic compounds of the hull showed a high percentage of antioxidant capacity 48% *vs*. 34% of inhibition [31]. Antioxidant activity has been thoroughly studied on the basis of a wide variety of methods such as: reducing/antioxidant power (FRAP) and (DPPH) radical scavenging check for the evaluation of reducing power and correlating them with the total phenolic resin content with antioxidant activity due to different methods. Furthermore, phenolic compounds are universally distributed and have been a great source for chemical, biological, agricultural and medical studies. Our present study suggests that it could be a very inexpensive source of new anti-multidrug resistant and antimicrobial treatments for skin pathogens, thus warranting further investigation.

Coconut shells were analyzed for their fatty acids using gas chromatography (GC) combined with mass spectrometry (GC–MS). The results have been presented in Table 1 saturated fatty acids and 15 unsaturated fatty acids were identified. There was a minimum and maximum percentage of *Cocos nucifera* saturated fatty acids present in the acid range of tetracosgnoic (0.2%) and hexadecoric (5.43%) acid, unsaturated fatty acids upper and lower ranges found in gamma linolenic acid (0.27%), 9-enoic acid octadec (11.89%), and octadec 9-enoic acid (11.89%). Fatty acids are among the major building blocks of living cells, making lipid biosynthesis a potent goal for compounds with antibiotic or antiviral and antifungal properties. Fungi release enzymes which break down the keratin substance of the superficial layer of skin. Release of cell wall-degrading enzymes, (glucanases, chitinases, xylanases, pectinases and polygalacturonases) was compared for the proliferation of fungal skin infection. The fungal enzymes’ activities of acidic hydrolases were clearly greater than those of alkaline hydrolases, and are responsible for the growth of skin infection. This activity can thus be correlated with the strong potential of fatty acids.

The effect of the fatty acids’ linolenic acid, linoleic acid, erucic acid and oleic acid on the growth of the dermatophytes *Microsporum canis, Microsporum gypseum* and *Aspergillus* species were examined *in vivo* and *in vitro*. Tridecatriecnoate and palmitoleate exhibited activity against all of the dermal mycosis. However, acid red was used for mycelial growth of *Aspergillus* at 7.30 relative percentage whereas fatty acids gave significant reductions in mycelial growth of *Trichophyton* group and reduced production of enzymes due to biosynthesis potential of fatty acids. All of the fatty acids reduced all of the fungi’s enzyme production significantly in liquid culture, when added to the media at 100 microM. Behenic acid had no effect on fungal growth at any concentration examined. The antidermal activities exhibited by unsaturated fatty acids may be useful for alternative approaches to controlling important human pathogens, such as those examined in this study. The mechanism by which these antifungal effects were generated is not known but, due to the continuous need for new environmentally benign approaches to disease control, further work on the antifungal effects of fatty acids should be undertaken.

### 3.3. Antifungal Activities

**Figure 6:**
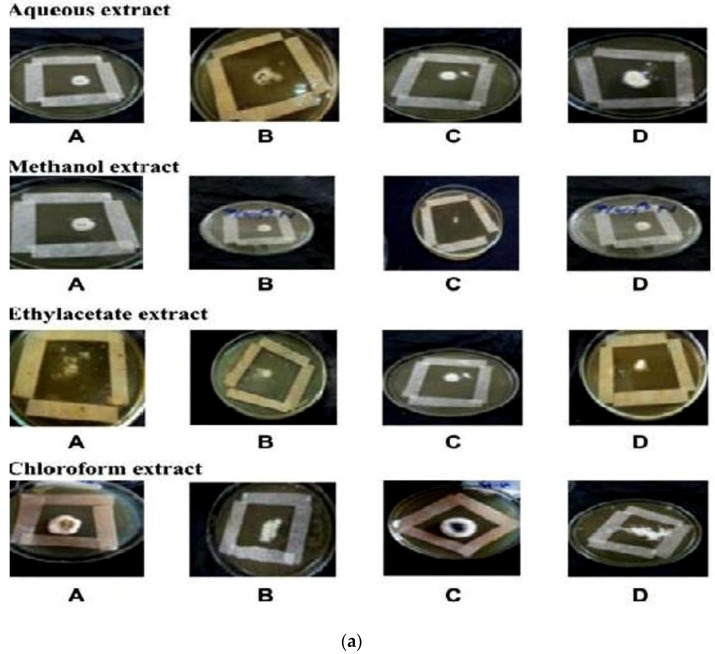

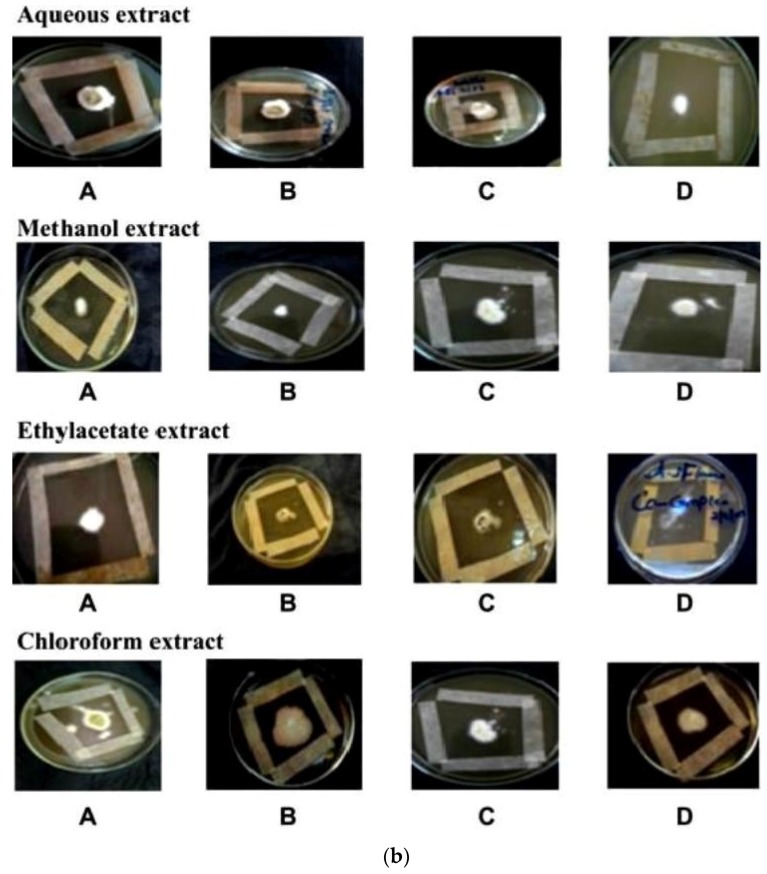
(**a**) An example of zones of inhibition produced from extracted thyme of shell extract of *Cocos nucifera* with different solvents after 16 h (aqueous extract, methanol extract, ethyl acetate extract and chloroform extract) on test organisms (A) *Aspergillus niger* (B) *Aspergillus flavus* (C) *Aspergillus fumigatus* (D) *Trichophyton verrucosum*; (**b**) An example of zones of inhibition produced from extracted thyme of shell extract of *Cocos nucifera* with different solvents after 16 h (aqueous extract, methanol extract, ethyl acetate extract and chloroform extract) on test organisms (A) *Microsporum canis* (B) *Microsporum gypseum* (C) *Trichophyton mentagrophyte* (D) *Trichophyton rubrun*.

**Figure 7:**
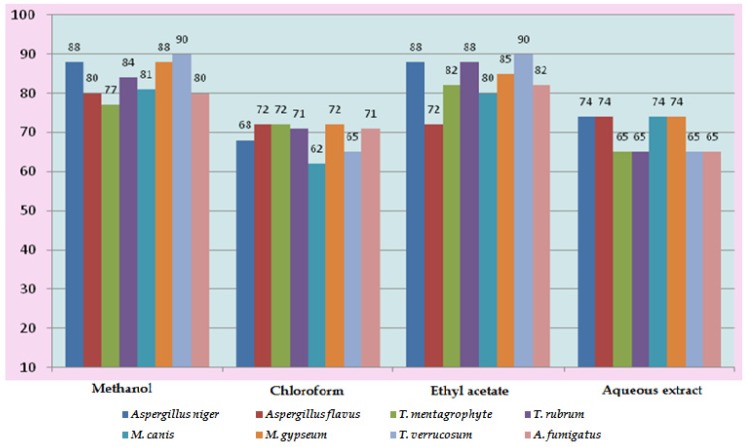
Minimum Inhibitory Concentration (MIC) of *Cocos nucifera.*

*In vitro* antifungal screening is generally performed by the strip method. This method is qualitative and trial indicating sensitivity or resistance of microorganism to the test material as fungi static or fungicidal activity of a coconut shell antioxidant infusion. The inhibitory effect of shell extract on the growth of various testing fungi is shown in Figure 6a,b. All four fractions of crude extract of *Cocos nucifera* showed activity against dermal mycosis and significant activity was exhibited in the different fractions of solvents. The highest inhibition activity was observed with methanol against test fungi *A. niger* 93% and *A. fumigatus* 91%, whereas moderate inhibition activity was found against *A. flavus* 87%, and *T. rubrum* 88%. The maximum inhibition activity was observed when ethyl acetate extract was used against *A. niger* 88% and *T. rubrum* 88%, respectively, while moderate inhibition activity was found against *T. mentagrophyte* 64% and *M. gypseum* 62.5%, respectively, and a minimum inhibitory effect with *A. flavors* 75% was measured. Maximum inhibition activity was observed with chloroform extract against *M. gypseum* 72.5% and *A. flavus* 72.5%, and moderate inhibition activity against *T. verrucosum* 65% and *A. fumigatus* 71.5%, respectively, was observed. Minimum inhibition activity against *M. cans* 62% was determined. The maximum inhibition activity was showed by aqueous extract against *A. niger*, *A. flavus*, *M. canis* and *M. gypseum* 74%, respectively, while moderate inhibition activity against *T. mentagrophyte* (65%) and *T. rubrum* (66%) and minimum inhibition activity against *Aspergillus* and *T. verrcosum* (64%) was observed. Crude extract of coconut shell was highly effective against both dermal mycoses tested with the MIC ranging from 1.4 mm to 2.145 mm. The zone of inhibition increased when increasing the concentration, and the inhibition effect was higher in methanol extract, chloroform extract and ethyl acetate extract, respectively. The average results were analyzed and are presented in Table 2 and Table 3. Minimum inhibitory concentration (MIC) values are shown in Figure 7.

## 4. Conclusions

Recent findings indicate the role of high phenolic compounds in antioxidant activity. Coconut shells can therefore be regarded as a promising candidate with high therapeutic potential for drug preparation. The current study may provide useful data concerning the different medicinal properties in coconut shells for protecting humans against common skin diseases. Since the shells of *Cocos nucifera* are discarded as waste—indeed comprising a major portion of the organic waste (agro-waste) of Pakistan—our study will open many research possibilities for the utilization of different agro-wastes as antifungal agents.

## Figures and Tables

**Table 1:** Saturated and unsaturated fatty acid of *Cocos nucifera* L. analyzed methyl ester.

S. No	Systematic Name	R.R.T	Common Name	Molecular Formula	Mol. wt	Rel.% ag
1.	*n*-Octanoate acid	24.12	Caprylate	C_9_H_18_O_2_	158	3.22
2.	Dodecanotic acid	22.70	Lauric acid	C_12_H_24_O_2_	200	0.89
3.	*n*-Hexadecanoic acid	29.80	Palmilate acid	C_17_H_34_O_2_	270	4.43
4.	*n*-Hexadecanoate	33.79	Margorate	C_6_H_12_O_2_	116	3.11
5.	*n*-Eicosanoic acid	39.92	Arachidic acid	C_32_H_64_O_2_	480	2.21
6.	Tetradeconic acid	45.66	Myristic acid	C_18_H_36_O_2_	284	5.33
7.	*n*-Octadecanoic acid	57.11	Stearic Acid	C_18_H_36_O_2_	284	3.84
8.	Tetracosanoic acid	63.66	Lignoceric Acid	C_24_H_48_O_2_	368	0.30
9.	*n*-Docosanoate	30.49	Behenic Acid	C_21_H_42_O_2_	326	4.29
10.	Hexadecanoic acid	35.7	Palmitic acid	C_19_H_38_O_2_	298	3.89
11.	*n*-Hexocosanoate acid	27.19	Cerotate	C_23_H_46_O_2_	354	0.48
12.	10-Octadecenoate	40.88	Oleate acid	C_20_H_40_O_2_	312	0.05
13.	Nonanoate	37.28	Laurate acid	C_21_H_42_O_2_	326	0.04
	**Total**					**32.08**
1.	*n*-Heptaecenoate	25.35	*n*-Heptaecenoate	C_17_H_32_O_2_	268	4.55
2.	Tridecatrienoate	26.8	Tridecatriecnoate	C_18_H_32_O_2_	280	7.30
3.	Methyl-2-Tridecynote	28.76	Decylacrylate	C_22_H_42_O_2_	338	5.34
4.	Methyl tricosenoate	33.76	Decylacrylate	C_14_H_26_O_2_	226	5.2
5.	2,4,5-Tetra decatriecnoate	34.39	Tetradecatrienoate	C_24_H_42_O_2_	362	4.48
6.	7-Ethyl-3-Methyl-2,6-undecadienoate	36.23	Undecadienoate	C_16_H_26_O_2_	250	4.37
7.	Pentadecatrienoate	38.27	Pentadecatrienoate	C_18_H_34_O_2_	282	3.89
8.	Hexadecadienoate	41.33	Hexadecadienoate	C_17_H_26_O_2_	262	5.50
9.	*n*-hexadecanoate	39.22	Plmitoleate	C_17_H_28_O_2_	264	5.27
10.	Heptadectrienoate	38.78	Heptadectrienoate	C_18_H_28_O_2_	276	3.22
11.	9,12,15,Octa decatrienoate	45.35	Octadecatrienoate	C_19_H_34_O_2_	294	3.13
12.	10-Octadecenoate	46.67	Oleate	C_19_H_36_O_2_	296	3.02
13.	*n*-Octadecanoate	48.49	Stearate	C_19_H_32_O_2_	292	4.6
14.	Eicosatrienoate	53.69	Eicosatrienoate	C_20_H_34_O_2_	306	4.83
15.	Methyl-17,18-hexacosenate	54.89	Hexacosenate	C_27_H_52_O_2_	408	3.21
	**Total**					**67.91**
	**13 Saturated, 15 Unsaturated = Total compounds 28 Total % of Saturated + Unsaturated Fatty Acid = 99.99%**					

Mol. wt = molecular weight; R.R.T = relative retention time; Rel.% age = relative percentage.

**Table 2:** *In vitro* Antifungal activity of *Cocos nucifera* against dermal mycosis.

Controlled reading in 72 h at 30 °C (mm)	*M. canis 35 mm*	*M. gypseum 40 mm*	*T. verrucosum 40 mm*	*A. fumigatus 35 mm*
Methanol inhibited reading at 30 °C after 72 h (mm). Inhibited (%)	10 mm 81	4 mm 88	4 mm 90	7 mm 80
Chloroform inhibited reading at 30 °C after 72 h (mm). Inhibited (%)	13 mm 62	11 mm 72.5	14 mm 65	10 mm 71
Ethyl acetate inhibited reading At 30 °C after 72 h (mm). Inhibited (%)	7 mm 80	6 mm 85	4 mm 90	8 mm 82
Aqueous inhibited reading at 30 °C after 72 h (mm). Inhibited (%)	4 mm 74	5 mm 74	14 mm 65	13 mm 65

**Table 3:** *In vitro* Antifungal activity of *Cocos nucifera* against dermal mycosis.

Controlled reading in 72 h at 30 °C (mm)	*A. niger 35 mm*	*A. flavus 40 mm*	*T. mentagrophyte 40 mm*	*T. rubrum 45 mm*
Methanol inhibited reading at 30 °C after 72 h (mm). Inhibited (%)	4 mm 88	8 mm 80	9 mm 77	5 mm 84
Chloroform inhibited reading at 30 °C after 72 h (mm). Inhibited (%)	11 mm 68	11 mm 72.5	11 mm 72	11 mm 71
Ethyl acetate inhibited reading at 30 °C after 72 h (mm). Inhibited (%)	8 mm 88	11 mm 72	7 mm 82	6 mm 88
Aqueous inhibited reading At 30 °C after 72 h (mm). Inhibited (%)	4 mm 74	5 mm 74	14 mm 65	13 mm 65
